# The kinetics of glutathione in the gastrointestinal tract of weaned piglets supplemented with different doses of dietary reduced glutathione

**DOI:** 10.3389/fvets.2023.1220213

**Published:** 2023-08-10

**Authors:** Yuhuang Hou, Joris Michiels, Céline V. Kerschaver, Mario Vandaele, Maryam Majdeddin, Els Vossen, Jeroen Degroote

**Affiliations:** Laboratory of Animal Nutrition and Animal Product Quality (LANUPRO), Department of Animal Sciences and Aquatic Ecology, Ghent University, Ghent, Belgium

**Keywords:** glutathione, kinetics, gastrointestinal tract, weaned piglet, different doses

## Abstract

This study aimed to investigate the kinetics of dietary GSH in the gastrointestinal tract and the effect of GSH on the intestinal redox status of weaned piglets. Forty-eight piglets with an average age of 26 days and an average body weight of 7.7 kg were used in this study. The piglets were divided into three treatment groups including the control group with a basal diet (CON) and two GSH groups with a basal diet supplemented with 0.1% GSH (LGSH) and 1.0% GSH (HGSH), respectively. The basal diet did not contain any GSH. The experiment lasted for 14 days, with eight animals sampled from each group on d5 and 14. The parts of 0–5%, 5–75%, and 75–100% of the length of the small intestine were assigned to SI1, SI2, and SI3. The results showed that GSH almost completely disappeared from the digesta at SI2. However, no difference in the GSH level in mucosa, liver, and blood erythrocytes was found. The level of cysteine (CYS) in SI1 digesta was significantly higher in HGSH than CON and LGSH on d14, and similar findings were observed for cystine (CYSS) in SI3 digesta on d5. The CYSS level in HGSH was also significantly higher than LGSH in the stomach on d14, while no CYS or CYSS was detected in the stomach for control animals, indicating the breakdown of GSH to CYS already occurred in the stomach. Irrespective of the dietary treatment, the CYS level on d14 and the CYSS level on d5 and 14 were increased when moving more distally into the gastrointestinal tract. Furthermore, the mucosal CYS level was significantly increased at SI1 in the LGSH and HGSH group compared with CON on d5. Glutathione disulfide (GSSG) was recovered in the diets and digesta from the LGSH and HGSH group, which could demonstrate the auto-oxidation of GSH. It is, therefore, concluded that GSH supplementation could not increase the small intestinal mucosal GSH level of weaned piglets, and this could potentially relate to the kinetics of GSH in the digestive tract, where GSH seemed to be prone to the breakdown to CYS and CYSS and the auto-oxidation to GSSG.

## Introduction

Glutathione (GSH) is a tripeptide containing a γ-amide bond and a sulfhydryl group composed of glutamic acid, cysteine (CYS), and glycine and is present in almost every part of the body cell (1). As the main endogenous antioxidant, GSH works in the detoxification of electrophiles and the alleviation of oxidative stress mainly caused by reactive oxygen species (ROSs) and reactive nitrogen species (RNSs) (2, 3). GSH was reported to keep the redox status balanced by reducing or neutralizing ROSs. As a result, the oxidized form of GSH, glutathione disulfide (GSSG), is generated (4). The ratio of GSH/GSSG or GSH/(GSH + GSSG) was used by many researchers to reflect the redox status in cells and to relate this to critical processes such as cell proliferation and apoptosis (5, 6). GSH preserves this dynamic balance by carefully coordinating the “GSH cycle” (7–9). GSH is synthesized successively by the enzymes glutamate–cysteine ligase (GCL) and GSH synthetase (GS) using glutamate, CYS, and glycine as resources (10). As the degradation of GSH, GSH can be degraded by gamma-glutamyltranspeptidase (γ-GT) and dipeptidase, so GSH is also the reservoir for CYS (11). Furthermore, γ-GT is recognized in the first step of a series of reactions in its environment according to its role in the mediating cleavage of gamma-glutamyl bonds, followed by redox equilibrium modulations. Therefore, the activity of γ-GT can be an index in predicting the process of pathophysiology (9). In recent years, many other enzymes were also reported in addition to γ-GT for the function of GSH degradation (8, 12).

Weaning transition is considered a very stressful period in pigs' life because piglets must adapt to the new living environment, should mingle with pigs from different litters, and consume completely different diets (13–15). Oxidative stress can be induced by these abrupt changes. The inflammation, villus atrophy of the small intestine, and even animal mortality were reported to be related to oxidative stress because the redox status could be affected by ROSs and RNSs and followed by small intestinal dysfunction (16–18). For example, Degroote et al. (19) found a significant decrease in the GSH level upon weaning transition in erythrocytes, duodenal, and jejunal mucosa of piglets. Additionally, an overall defect in the small intestinal barrier function co-occurred with GSH redox imbalance was also found (20). There is increasing evidence that indicated the need for antioxidant support during the weaning period as piglets suffer from barrier dysfunction and villus atrophy during the weaning transition (21). These events seem to be associated with GSH depletion or degradation and GSH redox imbalance (22), potentially leading to impaired redox signaling pathways that interfere with the cell transition cycle and phosphorylation status of tight junction proteins (23, 24). In other studies, it was demonstrated that the increased levels of GSH were found in different organs after oral GSH administration in rats (25). Yabuki and Fukunaga (26) found the anti-oxidative effect of oral GSH administration in relieving post-ischemia neuronal cell death of mice by decreasing the levels of oxidative markers such as 4-hydroxy-2-nonenal (4-HNE) and 8-hydroxy-2-deoxyguanosine (8-OHdG). These results seem to demonstrate that supplementary GSH could alleviate oxidation. However, some researchers have questioned the effectiveness of GSH by supplementing dietary GSH in healthy humans as studies have shown no significant difference in GSH levels in plasma or blood cells (27). This indicated that current research does not offer us a complete understanding of the effects of dietary GSH supplementation nor is it clear how GSH levels change throughout the gastrointestinal tract and if oral supplementation could increase small intestinal mucosal GSH levels.

Therefore, the experiment of dietary GSH addition to the weaned piglets was designed to investigate the kinetics of GSH in the gastrointestinal tract and the distribution of GSH in intestinal and hepatic tissues and in blood erythrocytes.

## Materials and methods

### Experimental animals and design

One day before the start of this study, the suckling piglets were selected from a total of 101 animals, originating from eight different litters at a commercial farm. All piglets were the same breed (Piétrain × Topigs TN70) and had an age difference of maximum 4 days. From each litter, six piglets having a body weight (BW) close to the median litter weight were selected. This resulted in a selection of 48 healthy animals from the eight litters with an average age of 26 days and having a BW ranging from 6.10 to 9.46 kg. At weaning (d0), the animals were divided into three treatment groups differing in the type of weaner diet: (1) a basal diet without GSH supplementation (CON), (2) a basal diet with 0.1% GSH supplementation (LGSH), and (3) a basal diet with 1.0% GSH supplementation (HGSH). Allocation was done according to a randomized block design with stratification for BW, litter origin, and sex. Twelve pens were used and set as four blocks of three pens, with each block including the three treatments. The piglets were allocated into four blocks for the balance of litter origin. To accomplish this, six animals from each litter were divided into three different pens within a block based on BW and sex. Piglets had *ad libitum* access to feed and water. The ingredient composition and the calculated nutrient composition of the basal diet are shown in Table 1. The CON diet contained 1.0% SiO_2_ (Diamol, Franz Bertram GmbH, Hamburg, Germany) as a source of 4 mol/L HCl insoluble ash and was not supplemented with GSH. From this diet, the LGSH and HGSH diets were prepared by replacing the corn with 0.1 and 1.0% GSH, respectively. As a source of GSH, reduced GSH (C_10_H_17_N_3_O_6_S, CAS 70-18-8) at 98.4% purity was obtained from AXO Industry International (Leuven, Belgium) as a crystalline white powder. The BW was registered individually on d5 and 14.

**Table 1:** Ingredient and calculated nutrient composition of basal diet (as-fed basis, %).

**Items**	**Content**
**Ingredients**	
Barley	30.00
Wheat	20.15
Corn	18.00
Toasted full-fat soybeans	10.00
Dextrose	5.00
Soy protein concentrate	4.00
Potato protein	4.00
Soybean meal	0.55
Soybean oil	2.59
Limestone	1.18
Monocalcium phosphate	0.69
*L*-Lysine	0.46
*L*-Threonine	0.16
*DL*-Methionine	0.17
*L*-Valine	0.02
*L*-Tryptophan	0.06
Vitamin and mineral premix^a^	0.50
Sodium chloride	0.25
Sodium bicarbonate	0.21
Diamol^b^	1.00
Amasil^c^	1.00
Phytase (providing 1,000 FTU)	0.01
Total	100.00
**Calculated composition, %**	
Dry matter	89.52
Crude protein	17.5
Crude fat	5.97
Calcium	0.64
Phosphorus	0.46
Lysine	1.28
Methionine	0.47

a Premix providing per kg of diet: vitamin A (retinyl acetate), 10,000 IU; vitamin D_3_ (cholecalciferol), 2,000 IU; vitamin E (dl-α-tocopherol acetate), 40 mg; vitamin K_3_ (menadione), 1.5 mg; vitamin B_1_ (thiamine), 1.0 mg; vitamin B_2_ (riboflavin), 4.0 mg; niacin, 30 mg; D-pantothenic acid, 15 mg; vitamin B_6_ (pyridoxine-HCl), 1.5 mg; vitamin B_12_ (cyanocobalamin), 20 μg; folic acid, 0.4 mg; biotin, 0.05 mg; choline chloride, 150 mg; Fe (FeSO_4_.H_2_O), 100 mg; Cu (CuSO_4_.5H_2_O), 20 mg; Mn (MnO), 30; Zn (ZnSO_4_.H_2_O), 70 mg; I (KI), 0.7 mg; Se (Na_2_SeO_3_), 0.25 mg.

b Diamol: SiO_2_, source of 4 mol/L HCl insoluble ash.

c Amasil: Composed of formic acid/sodium formate/water with the ratio of 61.5%/20.5%/18.5%.

### Sample collection

On d5, two animals per pen were selected for sampling based on average daily gain (ADG) and the litter origin. The ADG of the animals that were selected was positive, and the litter origin of animals selected in each block was equal. The remaining 24 animals were sampled on d14, except one animal that suddenly died on d14 by an unknown cause. This piglet was not treated with antibiotics before this incident. All the data from this dead animal were excluded from the data analysis.

At sampling, the animals were euthanized by electrical stunning and dissected at the facilities of Ghent University, which are on campus, next to the animal facilities. Blood was collected by exsanguination into heparinized tubes containing 200 μl of 1 mM bathophenanthroline disulfonic acid (BPDS). Hereafter, 0.5 ml of non-clotted blood was transferred to a 2 ml vial and immediately centrifuged (3,000 g, 10 min), and the supernatant was subsequently removed. To the residue, 0.6 ml milliQ water and 100 μl 70% metaphosphoric acid were added, followed by intense vortex and centrifugation (3,000 g, 10 min). A portion of the resulting acid extract (0.5 ml) was added to a tube with 50 μl solution containing 15 mmol/L γ-glutamyl-glutamate (γ-Glu-Glu) as the internal standard. This process was repeated in duplicate for each animal. Samples were snap-frozen in liquid nitrogen and stored at −80°C for further redox status analysis.

Following exsanguination, the animal was dissected for tissue sample collection. The gastrointestinal tract was removed from the body of the pig, and the stomach, small intestine, caecum, colon, and rectum were dissected. The length of the small intestine was measured, and 0–5%, 5–75%, and 75–100% of the length of the small intestine were assigned as SI1, SI2, and SI3, respectively. The digesta from each segment (stomach, SI1, SI2, and SI3) were collected in disposable cups quantitatively. The digesta were homogenized, whereafter a subsample was collected into a 2 ml plastic tube for the analysis of the redox status. The rest of the digesta was weighed. This sample was stored at −20°C for dry matter (DM) measurement. At the position of 5 and 75% of small intestinal length, a 20 cm intestinal segment was taken and opened longitudinally with a sharp scissor on an ice-cold surface to expose the epithelial surface. The mucosal layer was harvested by gentle scraping of the epithelium using a glass slide. A piece of the liver tissue was harvested from a fixed position on the right lobe of the liver for each animal. The tissue was cut from the liver and collected into a 2 ml tube. The mucosal and liver samples were snap-frozen in liquid nitrogen and stored at −80°C for the quantification of the redox status.

### Laboratory analysis

#### DM measurement

The weight of the empty plastic cup (N) and cup with digesta sample (N1) were registered during sampling. The DM was analyzed by using the freeze-drying method (FreeZone^®^ bench top freeze dryer, VWR International bvba, B-3001 Leuven, Belgium). Therefore, the DM samples in the −20°C freezer were transferred to −80°C before lyophilizing. Samples were put into the machine without the lid on the plastic cup, and the dryer was set a 0.2 vacuum at −50°C. For each run, 42 h were needed to complete the drying process, whereafter the weight was registered as N2.

The calculation for dry matter percentage is as follows:


$$\begin{array}{l} \text{DM }\left(\text{\%}\right)\;=\;\left(\text{N}2-\text{N}\right)/\left(\text{N}1-\text{N}\right){\;}^{*}100\%. \end{array}$$

#### Disappearance determination and calculations

The disappearance was assessed using the indicator method with 4 mol/L HCl insoluble ash as a marker (28). In brief, the empty crucibles were prepared by burning in the muffle furnace at 650°C for 30 min. After cooling down, the weight of the crucibles was registered as M0. Next, 5 ± 0.1 g of digesta was weighed in duplicate (M) and dissolved into 100 ml 4 mol/L HCl followed by boiling for 30 min. Then, the content of the solution was transferred and the unsoluble fraction was retained in the ashless filter paper. The filter paper with content was dried in an oven and put in the crucibles and burned in the muffle furnace at 650°C for 6 h. The final weight of the filter paper together with crucible was weighed up as M1.

The calculation of acid-insoluble ash is as follows:


$$\begin{array}{l} 4\text{ mol}/\text{L HCl insoluble ash }=\;\left(\left(\text{M}1\;-\text{ M}0\right)\right)/\text{M }\times \;100\;\left(\text{\%}\right), \end{array}$$

where *M* = mass in g of sample used for the analysis, M0 = mass in g of the empty crucible, and M1 = mass in g of the crucible and sample after incineration.

The calculations of disappearance are as follows:


$$\begin{array}{l} \text{Disappearance }=\;1\;\left(\text{G digesta }\left(\text{nmol}/\text{g}\right)/\text{G feed }\left(\text{nmol}/\text{g}\right)\right)\\ \times \;\left(\text{M feed }\left(\text{\%}\right)/\text{M digesta }\left(\text{\%}\right)\right), \end{array}$$

where *G* digesta = content of the target substrate in digesta (nmol/g), G feed = content of the target substrate in feed (nmol/g), *M* feed = percent of acid insoluble ash in feed (%), and *M* digesta = percent of acid insoluble ash in digesta (%).

### Redox status analysis

The concentration of CYS, cystine (CYSS), GSH, and GSSG in the red blood cell, the liver, the mucosa of the intestine at 5 and 75% of small intestinal length, as well as their concentrations in the digesta of the stomach, SI1, SI2, and SI3 were measured according to the method from Jones et al. (29) with some modifications. First, tissue samples were prepared by weighing 1.0 g of the sample and homogenizing it with 10 ml 5% perchloric acid solution (1:10 w/v) containing 0.2 mol/L boric acid (5% PCA/BA). Thereafter, 5 ml of 5% PCA/BA was added to the sample in a plastic tube of 50 cm^3^ and then homogenized at 900 rpm with the Turax homogenizer. The mixer of homogenizer and the wall inside of the plastic tube was rinsed with another 5 ml 5% PCA/BA. Two replicates of each sample were performed. Then, this homogenate was centrifuged at 15,000 g at 4°C for 5 min. A portion of the resulting acid extract (0.9 ml) was added to a tube containing 100 μl γ-Glu-Glu internal standard solution, followed by vertexing. Similar procedures were followed to extract digesta samples but with (1:4 w/v) dilution congruent to the concentration of target substrate in digesta. A 1:3 dilution was performed on red blood cell samples by using a 5% PCA/BA solution.

Following the extraction, the samples were derivatized by transferring 600 μl (500 μl for erythrocyte samples) of the solution into a glass tube, and adding 120 μl of iodoacetic acid (IAA, 7.4 mg/ml). After 30 min incubation, the pH of the solution was adjusted to 9.0 ± 0.2 with 1 M KOH/tetraborate solution. The solution was incubated for 20 min, followed by adding 600 μl of 20 mg/ml dansyl chloride (200 mg dansyl chloride was dissolved into 10 ml acetone) solution. The solutions were vortexed and incubated for 16–26 h in the dark at room temperature. Next, 1 ml chloroform (CHCl_3_) was added to remove the unreacted dansyl chloride. The samples were centrifuged (5 min, 3,000 g) before the transfer of an aliquot of the upper aqueous layer to an autosampler vial while applying filtration by a filter with a pore size of 0.2 μm. The derivates were separated on an aminopropyl column with a 250 × 4.6 mm and 10 μm particle size of (Nucleosil 120-7 NH2, Macherey-Nagel GmbH & Co. KG, Düren, Germany) by reverse-phase high-performance liquid chromatography (HPLC). Chromatographic runs were performed with methanol/water (80%/20%) and acetate-buffered methanol (pH = 4.6) as mobile phases and with fluorescent measurement at 335 nm of excitation and 518 nm of emission. The injection volume used was 25 μl. Solvent gradient and HPLC flow rate were set according to Jones et al. (29). The unit of the results was expressed based on the weight of wet samples. In samples where the CYS, CYSS, GSH, or GSSG levels could not be quantified due to concentrations below the detection limit, the value was considered to be not a number and hence was not replaced by any arbitrary value. The detection limit was determined by testing a series of different concentrations of the stock solution of target substrates, and the smallest area of peak was distinguished from the absence of the analyte (30). The limit of detection amounted to 0.27 nmol/g, 1.59 nmol/g, 0.31 nmol/g, and 1.60 nmol/g for CYS, CYSS, GSH, or GSSG, respectively. The results of the tests not able to reach the limit of detection were considered as no detection.

### Feed analysis

The DM of the diet was determined by oven drying at 103°C to harvest the constant weight (31). Crude protein content was calculated by multiplying the total *N* by 6.25, and the total *N* content was determined by using the Kjeldahl method (ISO 5983-1, I) (32). Crude fat was extracted by using diethyl ether with a Soxhlet system (ISO 6492, I) (33). The calcium and phosphorus content was determined by ICP-OES (ISO 11885, I) (34). The amino acid composition of protein-bound amino acids was determined by HPLC (ISO 13903, I) (35).

### Statistics

All the data were statistically analyzed with IBM SPSS statistics version 22.0 (SPSS Inc., Chicago, USA). First, the normality of the data was explored using the Shapiro–Wilkinson test, and the Kruskal–Wallis non-parametric test was used for analysis if the data did not satisfy the normality test. Second, Levene's test was used to check the homogeneity of variance, and the Welch test was used when the data did not satisfy the homogeneity test. One-way ANOVA procedure followed by the Tukey *post-hoc* test was used for data analysis when the data pass the normality test and homogeneity test. Difference was considered significant when a *p*-value ≤ 0.05, and tendency was assumed when 0.05 < *p* ≤ 0.1. The data are shown as least square means with the same standard errors of the means (SEM). The figures were made by using GraphPad Prism 8.0.2 (Graph Pad Software, Inc., La Jolla, CA, USA). The interaction effects of different sites of the gastrointestinal tract and GSH treatments on GSH concentrations in digesta in the figure were performed by the two-way ANOVA procedure with SPSS.

## Results

### DM of gastrointestinal digesta and BW of piglets

**Figure 1:**
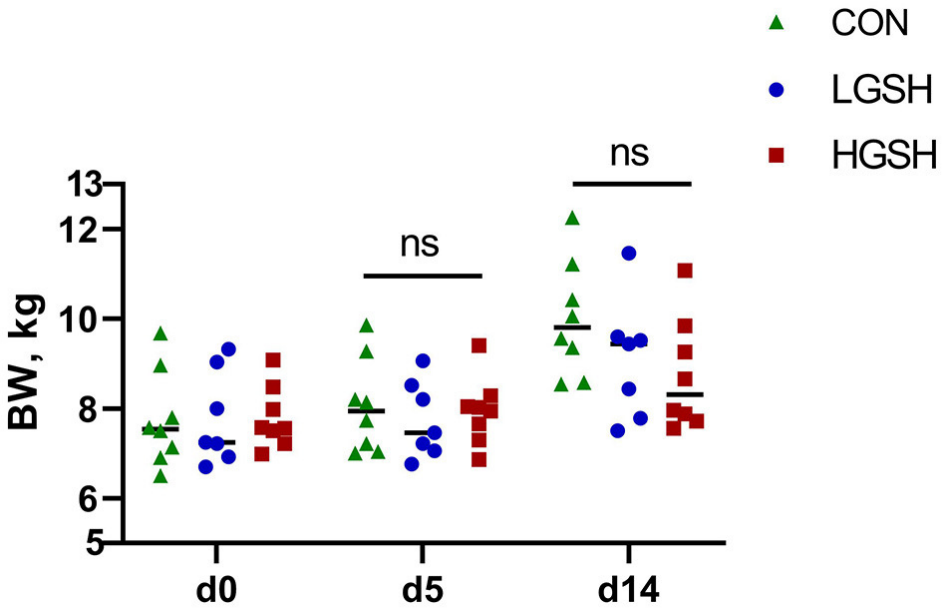
Effect of supplementary GSH on BW of piglets on d0, d5, and d14 from *ad libitum* fed basal diet (CON), basal diet with 1 g/kg GSH (LGSH), and basal diet with 10 g/kg GSH (HGSH; *n* = 7–8). BW, body weight; ns, no significance.

The results of DM of digesta from different gastrointestinal tract sites are shown in Table 2. No significant differences between the dietary treatments were found regarding the DM of the digesta content in all measured parts of the gastrointestinal tract. Similarly, DM content in the rectum at d5 and d14 was not different between treatments. BW of piglets is presented in Figure 1. No significant difference in BW was found between different groups.

**Table 2:** Dry matter (DM) of digesta in different gastrointestinal sites on d5 and d14 (*n* = 6–8).

**Item**		**Treatment**			**SEM**	***p*-value**
		**CON**	**LGSH**	**HGSH**		
Stomach	d5, %	28.2	27.9	27.5	0.60	0.469
	d14, %	29.3	29.7	26.4	0.67	0.089
SI1	d5, %	7.8	7.3	10.5	0.95	0.356
	d14, %	8.6	8.8	8.9	0.52	0.978
SI2	d5, %	7.8	8.2	9.6	0.75	0.606
	d14, %	8.2	8.0	8.5	0.63	0.956
SI3	d5, %	6.6	8.8	9.7	0.67	0.151
	d14, %	8.9	6.6	8.7	0.84	0.523
Caecum	d5, %	12.0	8.5	8.9	1.33	0.867
	d14, %	8.9	7.9	8.5	0.37	0.588
Colon	d5, %	17.6	12.3	14.0	1.39	0.300
	d14, %	17.1	13.3	15.6	1.00	0.146
Rectum	d5, %	27.3	20.2	20.1	2.27	0.336
	d14, %	21.7	19.3	21.9	1.62	0.798

CON, basal diet; LGSH, basal diet with 0.1% GSH supplementation; HGSH, basal diet with 1.0% GSH supplementation. SI1, digesta from 0 to 5% of length of the small intestine; SI2, digesta from 5 to 75% of length of the small intestine; SI3, digesta from 75 to 100% of length of the small intestine.

### Response of redox status in the digesta at different gastrointestinal sites

**Figure 2:**
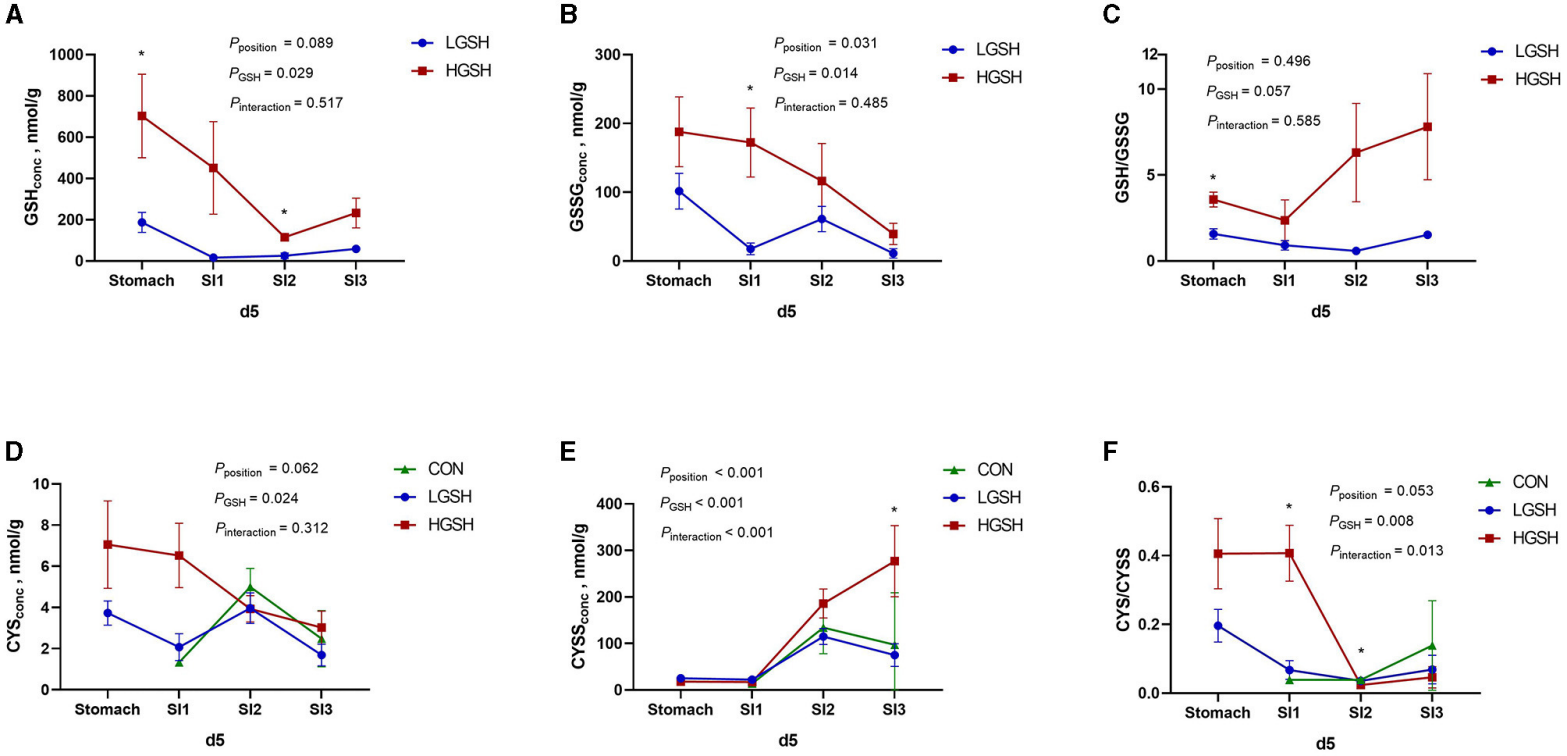
Glutathione and cysteine redox system in the gastrointestinal tract of piglets on d5 from *ad libitum* fed basal diet (CON), basal diet with 1 g/kg GSH (LGSH), and basal diet with 10 g/kg GSH (HGSH; *n* = 7–8). The results are presented as least squares means with SEM. Significance levels of main effects and interaction terms are indicated: *p*_position_ = the effect of different sites (stomach, SI1, SI2, and SI3) on reduced glutathione (GSH), oxidized glutathione (GSSG), cysteine (CYS), and cystine (CYSS) concentrations and ratios of GSH/GSSG and CYS/CYSS in the gastrointestinal tract across the other factors, *p*_GSH_ = the effect of GSH treatment on GSH, GSSG, CYS, and CYSS concentrations and ratios of GSH/GSSG and CYS/CYSS in the gastrointestinal tract across the other factor, *p*_interaction_ = the effect of interaction of different sites of the gastrointestinal tract and GSH treatment on GSH, GSSG, CYS, and CYSS concentrations and ratios of GSH/GSSG and CYS/CYSS in the gastrointestinal tract across the other factors. **(A)** GSH concentrations on d5; **(B)** GSSG concentrations on d5; **(C)** GSH/GSSG ratios on d5; **(D)** CYS concentrations on d5; **(E)** CYSS concentrations on d5; and **(F)** CYS/CYSS ratios on d5. ^*^Represents the significant difference among groups from the effect of GSH treatment (*p* ≤ 0.05).

**Figure 3:**
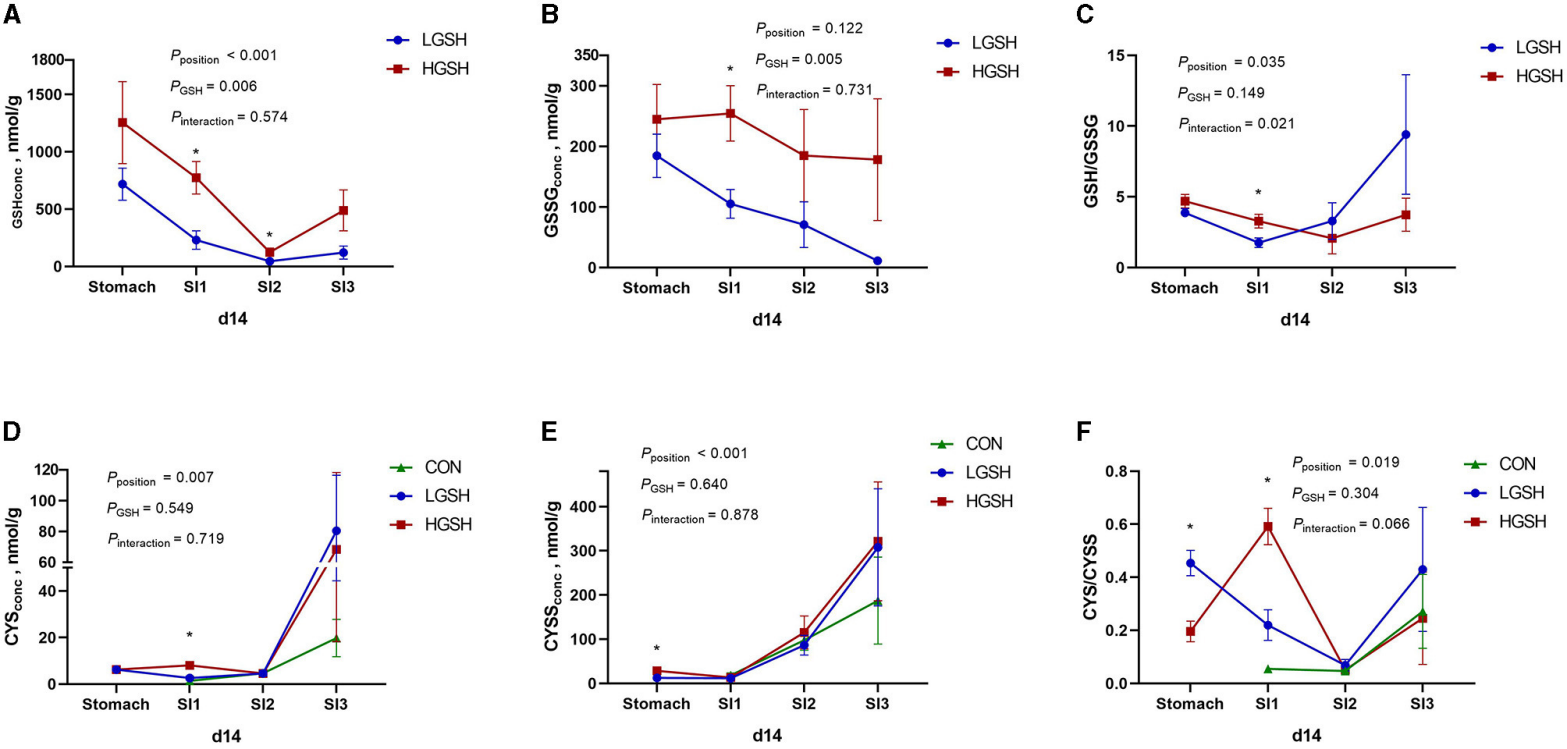
Glutathione and cysteine redox system in the gastrointestinal tract of piglets on d14, from *ad libitum* fed basal diet (CON), basal diet with 1 g/kg GSH (LGSH), and basal diet with 10 g/kg GSH (HGSH; *n* = 7–8). Results are presented as least squares means with SEM. Significance levels of main effects and interaction terms are indicated: *p*_position_ = the effect of different sites (stomach, SI1, SI2, and SI3) on reduced glutathione (GSH), oxidized glutathione (GSSG), cysteine (CYS), and cystine (CYSS) concentrations and ratios of GSH/GSSG and CYS/CYSS in the gastrointestinal tract across the other factors, *p*_GSH_ = the effect of GSH treatment on GSH, GSSG, CYS, and CYSS concentrations and ratios of GSH/GSSG and CYS/CYSS in the gastrointestinal tract across the other factors, *p*_interaction_ = the effect of interaction of different sites of the gastrointestinal tract and GSH treatment on GSH, GSSG, CYS, and CYSS concentrations and ratios of GSH/GSSG and CYS/CYSS in the gastrointestinal tract across the other factors. **(A)** GSH concentrations on d14; **(B)** GSSG concentrations on d14; **(C)** GSH/GSSG ratios on d14; **(D)** CYS concentrations on d14; **(E)** CYSS concentrations on d14; and **(F)** CYS/CYSS ratios on d14. *Represents the significant difference among groups from the effect of GSH treatment (*p* ≤ 0.05).

The concentrations of GSH, GSSG, CYS, and CYSS in digesta are shown in Table 3 and Figures 2, 3. No GSH and GSSG were found in the digesta from the CON group on d5 and d14 in either gastrointestinal site. The concentration of GSH in the stomach was significantly higher in the HGSH group as compared to the LGHS group (*p* = 0.039) on d5. This was not the case at d14 although numeric values are in the same line. Further down the intestinal tract, GSH levels in digesta were significantly higher in the HGSH group as compared to the LGSH group. This was the case for SI1 on d14 (*p* = 0.005) and SI2 on d5 (*p* = 0.016) and d14 (*p* = 0.037). A trend was observed in SI1 on d5 (*p* = 0.100). As no GSH was detected in SI3 on d5 in both CON and LGSH groups, it can also be stated that a dietary dose of 1.0% increased the GSH concentration of the digesta in this case. From Figure 2A, in the LGSH and HGSH groups, there was a trend for decreased GSH levels going from the stomach to SI3 on d5 (*p*_position_ = 0.089). A similar result was observed on d14, where GSH levels were significantly decreased from stomach to SI2 by 93.7% (*p* < 0.001, Figure 3A), irrespective of the dose. Similarly, the GSSG concentration in the digesta of SI1 was significantly higher in the HGSH group than the LGSH group on d5 (*p* = 0.029) and d14 (*p* = 0.011). This was not the case in the stomach, SI2 or SI3, although values seem to head in the same direction. Similarly, GSSG was also significantly decreased throughout the gastrointestinal tract (*p*_position_ = 0.014, Figure 2B) on d5 for treatment LGSH and HGSH, but this was, however, not the case at d14 (*p* = 0.122, Figure 3B). Regarding the ratio of GSH/GSSG in the digesta, GSH/GSSG in the HGSH group was significantly higher than the LGSH group in the stomach on d5 (*p* = 0.002) and in SI1 on d14 (*p* = 0.023). However, for the general treatment effect, it only showed a trend of increase in HGSH group on d5 (*p*_GSH_ = 0.057, Figure 2C) and the significant interaction effect on d14 (*p*_interaction_ = 0.013, Figure 3C). It is important to mention that the test of GSH in digesta from the caecum, colon, and rectum was also performed. However, the GSH was not found to be present in those samples.

**Table 3:** Level of GSH, GSSG, CYS, and CYSS in digesta in different gastrointestinal sites on d5 and 14 (*n* = 7–8)^1^.

**Item**		**Treatment**			**SEM**	***p*-value**
		**CON**	**LGSH**	**HGSH**		
**Stomach**						
d5	GSH, nmol/g	ND^2^	187.1^b^	702.9^a^	120.71	0.039
	GSSG, nmol/g	ND	101.5	187.9	29.67	0.152
	GSH/GSSG, (nmol/g)/(nmol/g)	ND	1.6	3.6	0.36	0.002
	CYS, nmol/g	ND	3.7	7.1	1.09	0.132
	CYSS, nmol/g	ND	25.5	18.3	3.24	0.292
	CYS/CYSS, (nmol/g)/(nmol/g)	ND	0.2	0.4	0.06	0.180
d14	GSH, nmol/g	ND	717.5	1,254.2	207.37	0.208
	GSSG, nmol/g	ND	184.5	244.5	34.66	0.408
	GSH/GSSG, (nmol/g)/(nmol/g)	ND	3.9	4.7	0.30	0.184
	CYS, nmol/g	ND	6.2	6.3	0.83	0.989
	CYSS, nmol/g	ND	12.5	28.5	2.98	0.003
	CYS/CYSS, (nmol/g)/(nmol/g)	ND	0.5	0.2	0.05	0.012
**SI1**						
d5	GSH, nmol/g	ND	16.2	451.2	141.90	0.100
	GSSG, nmol/g	ND	17.6	172.3	36.59	0.029
	GSH/GSSG, (nmol/g)/(nmol/g)	ND	0.9	2.4	0.71	0.193
	CYS, nmol/g	1.3	2.1	6.5	1.13	0.128
	CYSS, nmol/g	13.7	58.3	17.1	12.83	0.408
	CYS/CYSS, (nmol/g)/(nmol/g)	0.1^ab^	0.1^b^	0.4^a^	0.07	0.036
d14	GSH, nmol/g	ND	230.7	774.0	108.21	0.005
	GSSG, nmol/g	ND	105.2	254.3	31.80	0.011
	GSH/GSSG, (nmol/g)/(nmol/g)	ND	1.8	3.3	0.35	0.023
	CYS, nmol/g	1.4^b^	2.6^b^	8.0^a^	1.03	0.007
	CYSS, nmol/g	18.8	11.6	13.4	1.74	0.225
	CYS/CYSS, (nmol/g)/(nmol/g)	0.1^b^	0.2^ab^	0.6^a^	0.07	0.008
**SI2**						
d5	GSH, nmol/g	ND	25.1	115.1	18.76	0.016
	GSSG, nmol/g	ND	61.0	116.3	42.65	0.624
	GSH/GSSG, (nmol/g)/(nmol/g)	ND	0.6	6.3	2.34	0.380
	CYS, nmol/g	4.4	3.5	3.9	0.46	0.779
	CYSS, nmol/g	134.0	115.1	186.0	14.42	0.113
	CYS/CYSS, (nmol/g)/(nmol/g)	0.04^a^	0.04^ab^	0.02^b^	0.003	0.027
d14	GSH, nmol/g	ND	45.5	147.8	22.00	0.037
	GSSG, nmol/g	ND	70.8	184.7	44.26	0.216
	GSH/GSSG, (nmol/g)/(nmol/g)	ND	3.3	2.1	0.83	0.491
	CYS, nmol/g	4.7	4.6	4.5	0.65	0.997
	CYSS, nmol/g	97.8	86.7	115.3	16.71	0.784
	CYS/CYSS, (nmol/g)/(nmol/g)	0.1	0.1	0.1	0.01	0.631
**SI3**						
d5	GSH, nmol/g	ND	ND	232.5	65.61	ND
	GSSG, nmol/g	ND	11.1	39.4	10.41	0.306
	GSH/GSSG, (nmol/g)/(nmol/g)	ND	ND	7.8	0.76	ND
	CYS, nmol/g	2.9	1.7	3.0	0.57	0.398
	CYSS, nmol/g	97.4^b^	75.2^b^	277.1^a^	37.79	0.040
	CYS/CYSS, (nmol/g)/(nmol/g)	0.2	0.1	0.1	0.05	0.618
d14	GSH, nmol/g	ND	121.0	488.5	122.58	0.129
	GSSG, nmol/g	ND	11.1	178.0	63.35	0.219
	GSH/GSSG, (nmol/g)/(nmol/g)	ND	9.4	3.7	2.06	0.196
	CYS, nmol/g	19.8	80.4	68.3	20.57	0.516
	CYSS, nmol/g	187.3	307.9	321.3	68.20	0.692
	CYS/CYSS, (nmol/g)/(nmol/g)	0.3	0.4	0.3	0.10	0.869

1 Levels of CYS, CYSS, GSH, and GSSG in digesta were on a fresh matter basis.

2 ND: no detection. In the row of ND, the statistical analysis was performed by using the independent t-test. So only LGSH and HGSH groups were compared.

a, b Means in the same row without common superscript are significantly different (p ≤ 0.05).

CON, basal diet; LGSH, basal diet with 0.1% GSH supplementation; HGSH, basal diet with 1.0% GSH supplementation. SI1, digesta from 0 to 5% of length of the small intestine; SI2, digesta from 5 to 75% of length of the small intestine; SI3, digesta from 75 to 100% of length of the small intestine; GSH, reduced glutathione; GSSG, oxidized glutathione; CYS, cysteine; CYSS, cystine.

CYS and CYSS were not detected in the stomach from the CON group both on d5 and d14. However, as opposed to GSH and GSSG, CYS and CYSS gradually appeared from SI1 to SI3 for all treatments, thus Including CON (Figures 2D, 3D). On d5, CYS and CYSS were found from SI1 to SI3 in all groups but not in the stomach in the CON group. The concentration of CYSS was increased significantly in LGSH and HGSH groups along the gastrointestinal tract on d5 (*p* < 0.001, Figure 2E) and d14 (*p* < 0.001, Figure 3E), respectively. Similar to CYSS but on d14, CYS was also significantly increased in the LGSH and HGSH groups (*p* = 0.007, Figure 3D). The level of CYS in SI1 on d14 was higher in the HGSH group than in the CON and LGSH groups (*p* = 0.007). The concentration of CYSS in the HGSH group was significantly higher than in the CON and LGSH groups in SI3 on d5 (*p* = 0.040). Moreover, the CYSS level in the HGSH group was found to be significantly higher than the LGSH group in the stomach on d14 (*p* = 0.003). Interestingly, the ratio of CYS/CYSS in the HGSH group was significantly increased in SI1 on d5 (*p* = 0.036) and d14 (*p* = 0.008), while it was the lowest in SI2 on d5 (*p* = 0.027). An significant interaction effect of GSH and position on the ratio of CYS/CYSS was found on d5 (*p*_interaction_ = 0.013, Figure 2F) whilst only a significant position effect on d14 (*p*_position_ = 0.019, Figure 2F).

## Redox status in tissues and erythrocytes

The levels of GSH, GSSG, CYS, and CYSS, and their redox status in tissues and erythrocytes are shown in Table 4. The GSH concentration was not affected in the mucosa at 5% SI and 75% SI and in erythrocytes by adding dietary GSH both on d5 and d14. Only the concentration of GSSG in the LGSH group was significantly lower than the CON and HGSH groups in mucosa from 75% SI on d5 (*p* = 0.036). Regarding the redox status of GSH in the small intestine, the GSH/GSSG ratio was higher in the LGSH group as compared to the HGSH group in 5% SI on d5 (*p* = 0.021). In the liver, only the GSSG levels at d14 tended to be increased in the HGSH group as compared to the CON group (*p* = 0.067). Surprisingly, adding GSH to the diet did not result in a significant increase in the GSH level in the liver although numerical values seemed much higher at d14 when GSH was added to the diet (*p* = 0.451).

**Table 4:** Level of GSH, GSSG, CYS, and CYSS in different tissues and erythrocytes on d5 and 14 (n = 7–8).

**Item**		**Treatment**			**SEM**	***p*-value**
		**CON**	**LGSH**	**HGSH**		
**5%SI**						
d5	GSH, nmol/g	1,684.7	1,887.9	1,746.4	89.45	0.598
	GSSG, nmol/g	112.8	106.9	144.6	8.48	0.171
	GSH/GSSG, (nmol/g)/(nmol/g)	15.1 ab	18.3 a	11.4 b	1.06	0.021
	CYS, nmol/g	15.2 b	30.1 a	32.5 a	3.29	0.016
	CYSS, nmol/g	302.5	399.3	322.9	49.83	0.720
	CYS/CYSS, (nmol/g)/(nmol/g)	0.1	0.1	0.2	0.02	0.103
d14	GSH, nmol/g	1,651.5	1,873.8	1,836.4	107.99	0.817
	GSSG, nmol/g	128.4	142.0	145.8	10.41	0.657
	GSH/GSSG, (nmol/g)/(nmol/g)	14.2	21.7	13.0	3.33	0.954
	CYS, nmol/g	14.0	48.1	23.2	8.53	0.287
	CYSS, nmol/g	215.0	297.1	297.8	29.00	0.412
	CYS/CYSS, (nmol/g)/(nmol/g)	0.1	0.2	0.1	0.03	0.366
**75%SI**						
d5	GSH, nmol/g	1,634.9	1,349.1	1,564.3	109.38	0.560
	GSSG, nmol/g	153.5^a^	72.1^b^	155.2^a^	15.54	0.036
	GSH/GSSG, (nmol/g)/(nmol/g)	14.5	23.9	17.6	2.83	0.114
	CYS, nmol/g	29.4	49.7	62.3	8.92	0.431
	CYSS, nmol/g	755.8	956.6	863.7	108.99	0.505
	CYS/CYSS, (nmol/g)/(nmol/g)	0.1	0.1	0.1	0.01	0.842
d14	GSH, nmol/g	1,317.8	1,525.0	1,187.2	84.70	0.151
	GSSG, nmol/g	96.2	119.0	111.9	10.23	0.679
	GSH/GSSG, (nmol/g)/(nmol/g)	14.3	14.5	15.0	1.35	0.664
	CYS, nmol/g	40.1	50.5	85.4	9.20	0.149
	CYSS, nmol/g	649.1	557.2	984.5	79.12	0.059
	CYS/CYSS, (nmol/g)/(nmol/g)	0.1	0.1	0.1	0.01	0.132
**Liver**						
d5	GSH, nmol/g	3,327.3	3,540.2	3,532.9	167.07	0.887
	GSSG, nmol/g	212.8	98.1	117.7	38.44	0.332
	GSH/GSSG, (nmol/g)/(nmol/g)	29.6	86.0	33.6	15.59	0.334
	CYS, nmol/g	212.7	309.5	217.0	25.09	0.170
	CYSS, nmol/g	478.5	497.5	467.2	35.65	0.635
	CYS/CYSS, (nmol/g)/(nmol/g)	0.5	0.8	0.5	0.07	0.146
d14	GSH, nmol/g	4,074.9	4,906.5	5,992.1	540.22	0.451
	GSSG, nmol/g	269.3	253.9	468.0	42.99	0.067
	GSH/GSSG, (nmol/g)/(nmol/g)	41.3	29.5	17.8	8.48	0.379
	CYS, nmol/g	375.7	258.0	512.4	58.33	0.183
	CYSS, nmol/g	1,724.8	771.2	1,052.3	254.53	0.437
	CYS/CYSS, (nmol/g)/(nmol/g)	0.4	0.4	0.6	0.08	0.467
**Erythrocytes**						
d5	GSH, nmol/ml	293.9	245.5	336.4	19.69	0.214
	GSSG, nmol/ml	53.9	40.1	51.1	4.32	0.303
	GSH/GSSG, (nmol/ml)/(nmol/ml)	5.8	6.7	8.0	0.76	0.663
	d14	GSH, nmol/ml	328.5	296.3	348.6	13.69	0.124
		GSSG, nmol/ml	43.7	36.2	43.6	2.96	0.535
		GSH/GSSG, (nmol/ml)/(nmol/ml)	8.1	8.3	8.2	0.38	0.964

a, b Means in the same row without common superscript are significantly different (p ≤ 0.05).

CON, basal diet; LGSH, basal diet with 0.1% GSH supplementation; HGSH, basal diet with 1.0% GSH supplementation. 5%SI, mucosa from 0 to 5% of length of small intestine; 75% SI, mucosa from 75 to 100% of length of small intestine; GSH, reduced glutathione; GSSG, oxidized glutathione; CYS, cysteine; CYSS, cystine.

Different from GSH, the mucosal CYS levels were significantly increased at 5% SI in the LGSH and HGSH groups compared with the CON group on d5 (*p* = 0.016) with 98.1 and 113.9% of increment, respectively. This was not the case for other tissues and/or days. Next, CYSS concentrations in 75% SI tended to be higher for the HGSH group as compared to the CON and LGSH group on d14 (*p* = 0.059). No differences regarding the CYS or CYSS levels were observed in the liver tissue when adding GSH to the diet. No CYS and CYSS were detected in erythrocytes, and no treatment differences for the ratio of CYS/CYSS were found in tissues.

### Disappearance of GSH

The disappearance of GSH from the stomach to SI3 is shown in Table 5. The results are expressed as the disappearance and are joined with a number of replicates that document this result. For several animals, the amount of the digesta was too low to quantify the amount of acid-insoluble ash. Hence, a part of the data is missing, and therefore, no statistical test was performed to evaluate the treatment effect. It is important to mention that the GSH concentrations in feed were 813.2 nmol/g and 2,746.9 nmol/g in the LGSH and HGSH groups, respectively, and the GSSG concentrations in feed was 78.5 nmol/g and 164.0 nmol/g in the LGSH and HGSH groups, respectively. From the table, it can be deduced that GSH almost completely disappeared by SI2 in both LGSH and HGSH animals, with disappearance equaling 97.5 and 98.0% on d5, and 97.6 and 98.2% on d14, respectively. As previously stated, no GSH was found in the digesta of CON animals, and therefore, no disappearance could be computed. For animals at d5, most of the GSH seemed already to be disappeared from the stomach, and this net cumulative disappearance increased toward SI2. On d14, however, values appeared to be much lower in the proximal part of the digestive tract, where stomach levels amounted to 41.8%−69.0% and SI1 digesta reached 53.3%−79.1%, respectively, for treatment of LGSH and HGSH. It must be stressed that some of these values originate from a limited number of replicates, and this might explain why certain values are numerically higher in some proximal compartments of the digestive tract, while distal compartments still show a lower value.

**Table 5:** Disappearance of reduced glutathione (GSH) and total pool of GSH^a^ (GSH + GSSG) in the gastrointestinal tract of piglets on d5 and d14^b^.

**Item**		**Treatment**		
		**CON**	**LGSH**	**HGSH**
**Stomach**				
d5	GSH, %	ND^c^	84.0_n = 8_	80.5_n = 8_
	GSH + GSSG, %	ND	77.5_n = 8_	76.7_n = 8_
d14	GSH, %	ND	41.8_n = 6_	69.0_n = 8_
	GSH + GSSG, %	ND	32.9_n = 6_	65.0_n = 8_
**SI1**				
d5	GSH, %	ND	99.7_n = 1_	83.6_n = 4_
	GSH + GSSG, %	ND	99.3_n = 1_	79.86_n = 4_
d14	GSH, %	ND	79.1_n = 6_	53.3_n = 3_
	GSH + GSSG, %	ND	71.4_n = 5_	67.9_n = 2_
**SI2**				
d5	GSH, %	ND	97.5_n = 3_	98.0_n = 5_
	GSH + GSSG, %	ND	90.7_n = 2_	95.2_n = 5_
d14	GSH, %	ND	97.6_n = 6_	98.2_n = 6_
	GSH + GSSG, %	ND	96.2_n = 5_	95.6_n = 5_
**SI3**				
d5	GSH, %	ND	^*^	95.9_n = 2_
	GSH + GSSG, %	ND	99.9_n = 1_	96.0_n = 2_
d14	GSH, %	ND	^*^	89.5_n = 3_
	GSH + GSSG, %	ND	^*^	87.3_n = 3_

a GSH + GSSG was presented for the total pool of GSH.

b The results were expressed as disappearance, supported by the number of replicates. The statistical analysis was not performed according to the limited replicates of detection.

c ND, no detection.

* No result was harvested according to the low quantity of sample or low concentration of GSSG.

CON, basal diet; LGSH, basal diet with 0.1% GSH supplementation; HGSH, basal diet with 1.0% GSH supplementation. SI1, digesta from 0 to 5% of length of the small intestine; SI2, digesta from 5 to 75% of length of the small intestine; SI3, digesta from 75 to 100% of length of the small intestine; GSH, reduced glutathione; GSSG, oxidized glutathione.

The disappearance of the entire GSH pool, hence including both GSH and GSSG, is also presented in Table 5. It is shown that the total GSH pool most often showed a lower disappearance as compared to solely considering GSH although no statistics were executed. Perhaps the best example is the difference in the disappearance in the stomach, where the values for the total GSH pool ranged from 76.7 to 77.5% on d5, while GSH disappearance values amounted to 80.5%−84.0%. Similar findings can be observed in the stomach on d14.

## Discussion

### The kinetics of GSH in the gastrointestinal tract

GSH was reported to the protective effects against oxidative stress and intestinal barrier dysfunction. It was found that dietary GSH can enhance antioxidant capacity and mRNA expression of tight junction protein with the consequence of improvement in growth performance of weaned piglets challenged by diquat, especially at the dose of 100 mg/kg of supplementation (36). To disclose the distribution and concentration of exogenous GSH along with the gastrointestinal tract and tissues, the kinetics of dietary GSH was investigated by this study. In the current study, the concentration of dietary GSH tended to be decreased in GSH-supplemented animals when moving more distal into the gastrointestinal tract. The disappearance of GSH in SI2 from LGSH and HGSH groups was all over 97.5% no matter the number of doses or timepoint of the current study. In other words, supplementary GSH was almost completely absorbed or degraded before reaching the distal small intestine. This finding is similar to the results of the study by Hagen et al. (37), who found that GSH removal primarily occurred in the jejunum following GSH supplementation (5–50 mg/g). There seems to be a discrepancy in the kinetics between d5 and d14, specifically regarding the rate of GSH disappearance from the stomach. The rate of GSH depletion was found to be higher on d5 compared to d14. Potentially, the higher feed intake level of the animals at d14 compared to d5 resulted in a much higher daily intake of GSH and thus a less efficient removal of GSH from the lumen in the proximal parts of the gastrointestinal tract.

### Degradation and oxidation of GSH in the gastrointestinal tract

In the current study, CYS and CYSS were not detected in the stomach content of the CON group but can be detected in the LGSH and HGSH groups both on d5 and d14, which could indicate that CYS originates from GSH degradation in the stomach. Moreover, in the proximal part of the small intestine following the stomach (SI1), we also found a significant increase in the CYS level in digesta when animals were supplemented 1.0% GSH for a prolonged time (from d0 to 14), as well as a significant increase in the CYS/CYSS ratio of the HGSH group both on d5 and 14. The increase in CYS or CYSS here could be attributed to the action of the enzyme of γ-GT in degrading GSH (8, 38). The degradation consists of two parts that are spatially organized across the intestinal epithelium (38). Extracellular and luminal GSH can be cleaved by γ-GT and dipeptidase because of the presence of these two enzymes in the plasma and luminal apical membrane. This process generates the constituent amino acids of GSH, namely, CYS, glutamate, and glycine (39–41). After the absorption of CYS, glutamate, and glycine into the cytosol, GSH can be synthesized and even can be released again into the intestinal lumen for antioxidant protection (42). These processes are involved in the cycle can be called “GSH cycle,” where γ-GT plays a critical role in the regulation of redox metabolism. In addition to the hydrolysis and synthesis of antioxidants, this cycle also focuses on achieving redox equilibrium and effectively managing the metabolism of toxic-free radicals and xenobiotics (9).

In this study, it was interesting to notice that the ratio of CYS/CYSS in SI1 digesta was the highest in the HGSH group at d5 and 14, while on the other hand, the CYS/CYSS ratio was the lowest in SI2 digesta at d5. Although the levels of CYSS were not different according to the analysis among groups, the increase in CYSS from SI1 to SI2 can be observed. This indicated that the increased CYSS levels might originate from auto-oxidation of CYS, which easily takes place out of the cell under normoxic conditions (43, 44). According to the findings in the current study, a cycle could be found based on the mechanism of CYSS transport (45). In the intestinal tract, GSH is subject to degradation leading to the formation of CYS, which subsequently undergoes oxidation to generate CYSS. The resulting CYSS is transported across the epithelial cells of the small intestine, where it gets reduced before being released back into the intestinal lumen, thus completing the transport cycle. The uptake of CYSS was mainly mediated by a CYSS/glutamate antiporter. Once inside the intestinal epithelial cells, CYSS can be further metabolized or transported across the basolateral membrane into the bloodstream. The transport across the basolateral membrane is facilitated by other transporters, such as the neutral amino acid transporters (46). In consideration of methionine metabolism, the level of CYS and CYSS could be partly originated from methionine (47). However, the different profiles of CYS and CYSS levels between the treatments were undoubtedly related to the different GSH levels of the diets.

Given the fact that no GSH or GSSG was detected in the stomach and small intestinal digesta of control animals, the finding that GSSG was recovered from the gastrointestinal lumen in the LGSH and HGSH group could demonstrate the oxidation of GSH in the gastrointestinal tract. It seemed that the higher dose was less vulnerable to oxidation as this could be deducted for the higher GSH/GSSG ratio for HGSH animals at d5 in the stomach and d14 in SI1. However, this effect was not found for the other observations, leading to the conclusion that no clear effect of the dietary dose of GSH on the luminal GSH/GSSG ratio was observed here. Overall, the redox ratio of GSH/GSSG ranges from 0.58 to 9.41, disregarding the day of sampling or the intestinal compartment, which is much lower than the redox ratios measured in tissues. This indicates that a substantial part of the GSH pool is in an oxidized state in the gastrointestinal lumen, which is mainly related to the fact that intracellular levels of GSH and other components of the redox environment are more heavily regulated by the cell as compared to the redox environment of the extracellular space (48). Many studies reported that the small intestine suffers oxidative stress during the weaning transition (19, 20, 49). As a key antioxidant, GSH is crucial in the detoxification of xenobiotics and the elimination of ROS and RNS (50, 51). For example, an extracellular glutathione peroxidase isoform is reported to be secreted by intestinal cells into the lumen to take part in the protection of the intestinal mucosa against oxidant injury (52) and the removal of lipid oxidation products (53, 54). Therefore, GSH might be subjected to oxidation in the gastrointestinal tract, and this could be related to its protective function. Another cause for the conversion of GSH to GSSG could be the auto-oxidation of GSH to GSSG (55, 56). Interestingly, a study from Dahm and Jones (57) indicated that the jejunum can reduce luminal GSSG to GSH. This is attributed to the intracellular reduction of CYSS to CYS and the release of CYS in the lumen and luminal reduction of GSSG to GSH by CYS. These processes could function as a control of the luminal redox status. This is important for the absorption of redox-sensitive nutrients and the maintenance of gut health (57).

### Dietary GSH did not change the GSH level in the mucosa, liver, and erythrocytes

Previous research reported that piglets endure decreased GSH levels and redox imbalance following weaning (58). In this study, however, the GSH level in the proximal and distal small intestinal mucosa, the liver tissue, and erythrocytes was not affected by adding GSH to the diet at 0.1 and 1.0%. It was shown that GSH disappeared from the digesta in the current experiment, while previous rodent studies demonstrated an increase in GSH concentration in several tissues upon supplementation (25, 59, 60). For example, significant increases were found in the jejunum, lung, heart, liver, and brain after oral GSH administration to rats at 0.4, 1.0, and 4.0 mmol/kg of BW (25). Moreover, it was also demonstrated that intact GSH can be transported across intestinal epithelial cells and the initial uptake of GSH into cells is a rapid process (61). However, these studies were based on the mouse and rat model, which might not apply to the pigs. Hinchman and Ballatori (62) studied the GSH degrading capacities of the liver and the kidney in six different mammalian species and found a low γ-GT enzyme activity ratio of liver to kidney in rats while finding a high ratio in the pig. Since γ-GT is the enzyme in the first step of the degradation of GSH, a higher ratio of liver to kidney for γ-GT activity demonstrates the relative contribution of the liver in catabolizing GSH and GSH conjugates in the pig. In our study, we found no proof for increased metabolization of GSH in the liver. The hepatic GSH levels were not found to be significantly different among treatments although numeric values seemed much higher in LGSH and HGSH groups. The hepatic level of GSSG did tend to be increased on d14 by adding 1.0% GSH dietary, but the CYS and CYSS concentrations, on the other hand, were not significantly affected by the dietary treatment.

Previous research also demonstrated that species differences can be expected with respect to the activity of γ-GT enzyme in the intestinal mucosa (63). Rats, for example, were shown to be much more effective in absorbing GSH from the intestinal lumen than humans, and this was attributed to the relatively high expression of the γ-GT enzyme at the small intestinal brush border in humans (39, 63, 64) as this enzyme initiates the cleavage of GSH (65). Witschi et al. (66) further showed that GSH and its constituent amino acids CYS and glutamate were not increased within 270 min in plasma following the oral administration of single dose of 3.0 g of GSH to humans, suggesting that the systemic availability of GSH is negligible in man. However, GSH in blood is predominantly present in red blood cells (29, 67), and erythrocyte levels could therefore be expected to be affected as well. Nevertheless, in the study by Allen and Bradley (68), the levels of GSH, GSSG, and ratio of GSH/GSSG in erythrocytes were not improved by orally supplementing 1.0 g/day GSH to humans. Hence, it is plausible that, similar to man, GSH in blood erythrocytes and tissues are unlikely to be affected by oral GSH supplementation in pigs due to a high rate of first-pass splanchnic metabolism (69).

As a result of the extensive degradation of GSH, there was a notable rise in both CYS and CYSS levels in GSH-treated groups. CYS, as the indispensable sulfur-containing amino acid, serves various important functions such as participating in the protein structure, bolstering antioxidant defenses, and aiding in detoxification processes (43). Therefore, extensive investigations into the effects of dietary CYS have been conducted in order to attain a more profound understanding of its potential implications. It was demonstrated that intestinal absorption is the major metabolic fate of dietary CYS, with 75% of intake, 20%−25% of gut utilization, and 53% of the splanchnic first-pass uptake (69, 70). However, with the high ratio of uptake and utilization of dietary CYS, the level of GSH in tissue and blood was still not changed in the current study. Significantly, it has been discovered that the CYSS/glutamate antiporter system operates independently of intracellular GSH redox status instead of responding to the availability of extracellular CYSS (71). The CYSS/glutamate antiporter system seemed to be supported by our results as the system may target to reduce the excess of CYSS and rescue the extracellular CYS/CYSS redox status.

## Conclusion

In summary, the level of GSH in digesta was increased in different sites of the gastrointestinal tract by supplementing dietary GSH. Nevertheless, supplementing the diet of weaned piglets with 0.1% or 1.0% GSH did not change the level of GSH in mucosa of the small intestine, liver tissue, and blood erythrocytes. It is remarkable to find that GSH was not detected in the stomach and small intestinal digesta of unsupplemented animals, and an almost complete degradation or absorption of luminal GSH was observed when the distal small intestine is reached in supplemented animals. Decreased GSH levels could be explained by the breakdown of GSH to CYS and the auto-oxidation of CYS to CYSS as well as the oxidation of GSH to GSSG.

## Data availability statement

The original contributions presented in the study are included in the article/Supplementary material, further inquiries can be directed to the corresponding author.

## Ethics statement

The animal study was reviewed and approved by European Directive 2010/63/EU Belgian royal decree KB29.05.13.

## Author contributions

YH, JD, CK, and JM: conceptualization. YH, JD, EV, CK, MV, MM, and JM: methodology and writing—review and editing. YH, JD, and MM: software. YH, JD, and JM: validation. JD and JM: formal analysis and supervision. YH, EV, and JD: investigation. CK and JM: resources. YH and JD: data curation. YH: writing—original draft preparation and project administration. JM: funding acquisition. All authors have read and agreed to the published version of the manuscript.
