# Supplementary material for: The kinetics of glutathione in the gastrointestinal tract of weaned piglets supplemented with different doses of dietary reduced glutathione
Source: Front Vet Sci. 2023 Aug 10;10:1220213. doi: 10.3389/fvets.2023.1220213 (PMC10448897; doi:10.3389/fvets.2023.1220213)
Supplement: Supplementary file 1 [file Table_1.docx]

Table S1. Effects of dietary reduced glutathione (GSH) supplementation on growth performance in weaned piglets (n = 8).

| Item | |  | TREATMENT | | |  | SEM |  | *P*-value |
| --- | --- | --- | --- | --- | --- | --- | --- | --- | --- |
|  | |  | CON | LGSH^3^ | HGSH |  |  |  |  |
| Body weight (BW) ^1^ | | | | | |  |  |  |  |
|  |  | d0, kg | 7.7 | 7.7 | 7.8 |  | 0.12 |  | 0.961 |
|  | | d5, kg | 8.0 | 7.6 | 7.9 |  | 0.13 |  | 0.290 |
|  | | d14, kg | 10.0 | 9.1 | 8.8 |  | 0.28 |  | 0.161 |
| Average daily gain (ADG)^2^ | | | | | |  |  |  |  |
|  | | d0-5, g/d | 60.5 ^a^ | -35.9 ^b^ | 23.8 ^ab^ |  | 15.14 |  | 0.010^1^ |
|  | | d5-14, g/d | 215.6 ^a^ | 150.2 ^ab^ | 89.7 ^b^ |  | 20.35 |  | 0.027 |
|  | | d0-14, g/d | 160.2 | 94.9 | 67.7 |  | 16.63 |  | 0.051 |
| Average daily feed intake (ADFI) | | | | | |  |  |  |  |
|  | | d0-5, g/d | 147 | 83 | 121 |  | 11.6 |  | 0.053 |
|  | | d5-14, g/d | 305 | 249 | 236 |  | 20.5 |  | 0.384 |
|  | | d0-14, g/d | 222 | 161 | 175 |  | 13.8 |  | 0.178 |
| Average daily water intake (ADWI) | | | | | |  |  |  |  |
|  | | d0-5, mL/d | 574 | 499 | 658 |  | 30.4 |  | 0.116 |
|  | | d5-14, mL/d | 1211 | 769 | 1478 |  | 190.3 |  | 0.276 |
|  | | d0-14, mL/d | 983 | 673 | 1185 |  | 132.5 |  | 0.257 |
| Feed conversion ratio ^4^ (FCR) | | | | | |  |  |  |  |
|  | | d0-5 | 1.0 | -0.4 | 0.1 |  | 0.26 |  | 0.070 |
|  | | d5-14 | 0.7 | 0.6 | 0.3 |  | 0.11 |  | 0.421 |
|  | | d0-14 | 0.7 | 0.5 | 0.3 |  | 0.10 |  | 0.289 |

^1^ The replicate of BW on d0 and d5 was 16, all the animals were registered and took into the average calculation (n = 16). Half animals were slaughtered on d5, so the replicate of BW on d14 was 8 (n = 8).

^2^ ADG were calculated based on pen level, so the replicate here was 4 (n = 4).

^3^ One dead animal from LGSH group, the replicate here on d14 was 7 (n = 7).

^4^ Feed conversion ratio = average daily gain /average daily feed intake.

^a−b^ Means in the same row, different superscript letters show significant difference (*P* < 0.05).
